# The effect of respiratory muscle training on swimming performance: a systematic review and meta-analysis

**DOI:** 10.3389/fphys.2025.1638739

**Published:** 2025-07-17

**Authors:** Shunfang Liu, Pengpeng Gou, Menglong Lin

**Affiliations:** ^1^Faculty of Sport and Leisure, Guangdong Ocean University, Zhanjiang, China; ^2^Program in Global Exercise Science, Arts & Sports College, Inha University, Incheon, Republic of Korea

**Keywords:** respiratory muscle training, inspiratory muscle training, swimming performance, athletic adaptation, meta-analysis

## Abstract

**Introduction:**

Respiratory muscle training, which targets the inspiratory and/or expiratory muscles to enhance respiratory efficiency, is recognized as a method for improving athletic performance; however, its effectiveness in enhancing swimming performance remains controversial. This study aimed to evaluate the effects of respiratory muscle training on swimming performance.

**Methods:**

Methodology followed the PRISMA guidelines. A comprehensive literature search was conducted in eight databases (Web of Science, PubMed (comprising MEDLINE and PubMed Central), SPORTDiscus, ScienceDirect, Scopus, Cochrane Library, Embase, and ProQuest) and supplemented with manual searches of other sources (e.g., Google Scholar) up to 22 May 2025. Studies were eligible for inclusion if they met the following criteria: (a) participants were healthy individuals without diagnosed disease or disability; (b) the intervention involved respiratory muscle training compared to a sham or control condition in a randomized controlled trial or controlled clinical trial; (c) swimming performance was reported as an outcome with sufficient data to compute effect sizes; and (d) the full text was available.

**Results:**

Results of this systematic review revealed that of the 1,044 articles retrieved from the search strategy, 10 met the inclusion criteria. Meta-analysis indicated that respiratory muscle training significantly improved swimming performance, with low heterogeneity and no evidence of publication bias. Among the included studies, respiratory muscle training protocols were typically conducted at 50%–80% of maximal inspiratory pressure for 6–8 weeks, with a frequency of 3–14 sessions per week. However, substantial variability in training frequency, progression, and duration limited direct comparisons between interventions. Due to inconsistent and limited reporting, subgroup analysis based on gender, stroke style, or competitive level could not be performed.

**Discussion:**

Respiratory muscle training appears to be an effective adjunct to swimming training, contributing to improved performance. Future studies should prioritise protocol standardisation, elucidate the dose-effect relationship, and explore moderating factors such as gender, stroke type, and training status. Registered on PROSPERO (registration number: CRD420251051091).

**Systematic Review Registration:**

Identifier CRD420251051091.

## 1 Introduction

Swimming performance relies on the coordinated function of multiple physiological systems, with respiratory efficiency being a crucial yet often overlooked limiting factor. Swimmers face several unique physiological challenges in the aquatic environment. First, hydrostatic pressure causes elastic loading of the thoracic cavity, increasing the work and energetic cost of breathing, thereby elevating the burden on the inspiratory muscles (Pendergast et al., 2015). Second, the biomechanics of the stroke constrain the rhythm and timing of breathing, forcing it to adapt to the stroke cycle (Seifert et al., 2010). Additionally, during submersion, the diaphragm and respiratory muscles generate less force in the supine position than in the upright posture (Lomax and McConnell, 2003). These factors increase the likelihood of respiratory muscle fatigue, which can trigger the respiratory metaboreflex—leading to vasoconstriction in inactive limbs (St Croix et al., 2000), a 23%–30% reduction in limb blood flow, and increased vascular resistance (Sheel et al., 2001). In swimmers, these physiological constraints can impair stroke efficiency, elevate energy expenditure, and reduce overall training capacity. For instance, inspiratory muscle fatigue may lead swimmers to adopt suboptimal breathing patterns or shorten their stroke cycles to accommodate respiratory demands (Seifert et al., 2010; Lomax and Castle, 2011), both of which can negatively impact performance metrics such as sprint time and distance per stroke.

Respiratory muscle training (RMT), including inspiratory muscle training (IMT), expiratory muscle training (EMT), combined, or breath-holding protocols, aims to improve respiratory endurance and/or strength through resistance-based (e.g., pressure-threshold loading) or endurance-based strategies (e.g., voluntary isocapnic hyperpnea using devices like Spirotiger) (McConnell and Romer, 2004; McConnell, 2013). A typical RMT protocol involves training using pressure-threshold devices at 50%–70% of maximal inspiratory pressure (MIP), performed twice daily, with 30 effective breaths per session, 6–7 days per week, for at least 4–6 weeks (McConnell, 2013). Over the past decades, the role of RMT in enhancing athletic performance has been extensively investigated. For instance, in land-based endurance sports such as cycling and running, multiple studies have shown that RMT significantly improves time trial performance and delays fatigue (Holm et al., 2004; Romer et al., 2002; Bailey et al., 2010; Archiza et al., 2018). In combat sports, Tosun et al. reported that RMT significantly improved respiratory muscle strength and aerobic endurance in young wrestlers (Tosun et al., 2025).

Swimming is no exception. Several studies have reported that RMT can significantly improve swimming performance (Jakubovskis et al., 2024; Ohya et al., 2021). The proposed physiological mechanisms may include: enhanced inspiratory muscle strength, enabling swimmers to overcome hydrostatic pressure on the thoracic cavity, reduce the energy cost of breathing, and redirect metabolic resources toward propulsion (Pendergast et al., 2015; Gómez-Albareda et al., 2023); improved respiratory muscle function, allowing for more efficient breathing within constrained stroke cycles, minimizing movement disruption, and optimizing stroke frequency and length (Kilding et al., 2010); targeted training that increases diaphragmatic force output efficiency in a supine aquatic position (Lomax and McConnell, 2003); and greater respiratory muscle fatigue resistance, which may attenuate sympathetically mediated respiratory metaboreflexes, preserve limb blood flow and vascular function, and delay peripheral fatigue during exercise (St Croix et al., 2000; Sheel et al., 2001; Kowalski et al., 2022). However, other studies have failed to observe significant improvements in swimming performance following RMT (Cunha et al., 2019; Wells et al., 2005). These inconsistencies may be attributed to methodological limitations, including lack of randomisation, small sample sizes, and variations in RMT protocols, all of which limit cross-study comparability. For example, Cunha et al. implemented a 12-week program involving 30 inspiratory efforts twice daily, 5 days per week, at 50% of MIP in 32 elite swimmers (Cunha et al., 2019). In contrast, Jakubovskis et al. conducted a 4-week intervention consisting of modified respiratory exercises three times per week with 31 swimmers (Jakubovskis et al., 2024). Given these methodological differences, a meta-analytic synthesis is warranted to enhance the scientific rigor of RMT-based training recommendations for swimmers.

Previous reviews, including that by HajGhanbari et al., have focused on endurance sports such as football, cycling, and rowing, and confirmed RMT’s positive effects on endurance capacity, pulmonary function, and muscle strength (HajGhanbari et al., 2013; Xavier et al., 2025; León-Morillas et al., 2021; Illi et al., 2012). However, to date, no meta-analysis has systematically evaluated the effects of RMT on swimming performance while assessing the quality and risk of bias of included studies. One study by Tello et al. showed improvements in MIP in both elite and non-elite swimmers but did not assess direct performance outcomes (Carvajal-Tello et al., 2024). Therefore, this meta-analysis included randomized controlled trials (RCTs) or controlled clinical trials (CCTs) to investigate the effects of RMT on swimming performance in healthy individuals. Based on the PICO framework, the review focused on: Population: Healthy individuals or competitive swimmers; Intervention: RMT, IMT and/or EMT; Comparison: No RMT or placebo intervention; Outcome: Swimming performance outcomes.

## 2 Methods

This study adhered to the Preferred Reporting Items for Systematic Reviews and Meta-Analyses (PRISMA) guidelines (Page et al., 2021).

### 2.1 Eligibility criteria

Studies were included if they met all of the following criteria: (1) Participants were healthy and able-bodied individuals of any age, with no diagnosed diseases or physical disabilities; (2) the study design was a RCT or CCT; (3) the experimental group imposed additional respiratory muscle-related training, whereas the control group was identical to the experimental group except that they did not impose additional respiratory muscle-related training (or a placebo was imposed); (4) at least one swimming performance-related outcome was reported, and sufficient data were available to calculate effect sizes (ES); and (5) the full text was accessible.

### 2.2 Information sources

An independent researcher conducted a Boolean search across eight databases—Web of Science, PubMed (comprising MEDLINE and PubMed Central), SPORTDiscus, ScienceDirect, Scopus, Cochrane Library, Embase, and ProQuest. It is important to note that the search strategy for this study was expanded beyond the scope initially registered in PROSPERO, in order to broaden the retrieval of relevant literature. The search was conducted without restrictions on language, publication type, or publication date, with the final search performed on 22 May 2025. In addition, supplementary sources such as Google Scholar and ResearchGate were consulted, and the reference lists of relevant articles were manually screened to identify additional eligible studies.

### 2.3 Search strategy

The search terms are (‘Breathing Exercises’ OR ‘Exercise, Breathing’ OR ‘Respiratory Muscle Training’ OR ‘Muscle Training, Respiratory’ OR ‘Training, Respiratory Muscle’ OR ‘Inspiratory Muscle Training’ OR ‘Expiratory Muscle Training’) AND (‘Swimming Exercise’ OR ‘Swimming Performance’ OR ‘Swimming’). Detailed search strategies are provided in Supplementary Appendix A. Two independent reviewers imported the retrieved records into EndNote X9 for reference management and duplicate removal. Titles and abstracts were then screened independently based on the predefined eligibility criteria. All reviewers underwent standardized training prior to the screening process to ensure consistency. After the initial screening, the full texts of potentially eligible studies were assessed against the inclusion criteria. In cases of disagreement between the two reviewers, a third researcher was consulted to resolve discrepancies and reach consensus.

### 2.4 Risk of bias

In accordance with the PROSPERO registration protocol, the risk of bias of included randomized controlled trials was assessed using the Cochrane Risk of Bias 2.0 (RoB 2) tool (Higgins et al., 2024). Two independent reviewers conducted the assessments. This domain-based tool evaluates five specific bias domains: (1) bias arising from the randomization process, (2) bias due to deviations from intended interventions, (3) bias due to missing outcome data, (4) bias in measurement of the outcome, and (5) bias in selection of the reported result. Each domain contains a number of signalling questions. Once the signalling questions are answered, the next step is to reach a risk-of-bias judgement and assign one of three levels to each domain: ‘Low risk of bias’, ‘Some concerns’, or ‘High risk of bias’ (Higgins et al., 2024). Disagreements between the two reviewers were resolved through discussion with a third reviewer until consensus was reached.

### 2.5 Data extraction

Each study was coded based on the following variables: first author, participant characteristics (gender, age, swimming experience), intervention details for experimental and control groups, duration of intervention, and swimming performance outcomes (Table 1). Data for this study were independently extracted by two reviewers. Discrepancies were resolved through discussion, and a third reviewer was consulted to arbitrate any unresolved conflicts when necessary. Although inter-rater reliability statistics were not calculated, all discrepancies were resolved prior to data synthesis to ensure consistency and objectivity. Interventions in the experimental group included respiratory muscle-related approaches, such as IMT, EMT, and breathing exercises. Swimming performance was defined as the time taken to complete a specific swimming event. If baseline performance differed significantly between groups, the outcome was excluded from the analysis. In studies with three or more groups (e.g., two experimental and one control group), groups with incomplete data or protocol deviations (e.g., missing follow-ups) were excluded from the analysis. When studies presented swimming performance using bar charts with error bars without reporting exact means and standard deviations, data were extracted using digitization software by a designated researcher (Higgins et al., 2024).

**TABLE 1:** Basic characteristics of included studies in the meta-analysis (n = 10). M = mean, SD = standard deviation.

Study	Subjects	Sex	Age (M±SD)	Swimming experience (M±SD)	Experimental group/Control group treatment	Duration of intervention	Swimming performance index
Ohya et al. (2021)	20	EXP: 10M CON: 10M	EXP: 19 ± 1 year CON: 19 ± 1 year	EXP: 785 ± 53 FINA points CON: 762 ± 45 FINA points	IMT (50%-75%MIP)/no IMT	12 times per week of 6 weeks	100 m Freestyle
Kilding et al. (2010)	16	EXP: 5M3F CON: 5M3F	EXP: 19.1 ± 2.6 years CON: 19.0 ± 2.1 years	EXP: 7.8 ± 3.1 years CON: 8.1 ± 2.9 years	IMT (50%MIP)/sham IMT	14 times per week of 6 weeks	100 m/200 m/400 m (stroke not reported)
Wells et al. (2005)	34	14M20F	15.6 ± 1.3 years	at least 3 years	IMT (50%-80%MIP)/sham IMT	10 times per week of 12 weeks	200 m Freestyle
Lemaitre et al. (2013)	20	EXP: 6M4F CON: 7M3F	EXP: 16.5 ± 2.4 years CON: 16.1 ± 2.0 years	train for 45–48 weeks each year	RMT (40%–50% of vital capacity)/no RMT	5 times per week of 8 weeks	50 m/100 m (stroke not reported)
Kapus (2013)	12	EXP: 3M4F CON: 2M3F	EXP: 14 ± 1 year CON: 14 ± 1 year	at least 6 years	IMT (50%MIP)/sham IMT	14 times per week of 6 weeks	50 m Freestyle 50 m Butterfly 50 m Breaststroke 100 m Freestyle
Tan et al. (2023)	43	EXP: 20M CON: 23M	EXP: 21.21 ± 0.61 years CON: 21.26 ± 0.74 years	about 6 months	IMT/no IMT	3 times per week of 6 weeks	100 m Freestyle
Lomax et al. (2019)	33	18M15F	LOW: 16 ± 3 years HIGH: 16 ± 1 year	3–8 years	IMT (50% PImax )/no IMT	14 times per week of 6 weeks	100 m Freestyle 200 m Freestyle
Yañez-Sepulveda et al. (2021)	15	EXP: 9M CON: 6M	EXP: 15.5 ± 1.15 years CON: 14.7 ± 1.09 years	EXP: 7.0 ± 1.12 years CON: 6.0 ± 0.89 years	IMT (50%-70%MIP)/no IMT	14 times per week of 4 weeks	50 m Freestyle 100 m Freestyle
Bernhardt (2025)	17	15M2F	EXP: 19.6 ± 1.1 years CON: 19.1 ± 1.0 years	EXP: 10.6 ± 4.8 years CON: 10.5 ± 4.6 years	IMT (75–140%MEP)/sham IMT	5 times per week of 4 weeks	6*100 m Freestyle
Ghannadi et al. (2024)	20	EXP: 10M CON: 10M	EXP: 13.61 ± 1.51 years CON: 12.53 ± 2.56 years	at least 2 years	IMT (30%MIP-30%MIP+70cmH_2_O)/sham IMT	14 times per week of 8 weeks	50 m (stroke not reported)

### 2.6 Synthesis of results

In this study, Review Manager (RevMan) version 5.3 was used to analyze extracted data (sample size, mean, and standard deviation) for overall effect estimation (forest plot) and bias assessment (funnel plot). Stata version 17.0 was employed for sensitivity analysis. Given that baseline values were generally balanced across studies, post-intervention data were analyzed using a fixed-effects model. Heterogeneity, defined as the variability in effect sizes across studies, was assessed using the Chi^2^ p-value and the I^2^ statistic. A p-value >0.1 and I^2^ < 40% were considered indicative of low heterogeneity (Higgins et al., 2024). Because the included studies involved different swimming strokes and outcome measures, the standardized mean difference (SMD) was used for pooled analysis. A p-value <0.05 was considered statistically significant. SMD values were categorized as follows: trivial (<0.2), small (0.2–0.49), moderate (0.5–0.79), and large (≥0.8) (Cohen, 1988).

## 3 Results

### 3.1 Study characteristics

**FIGURE 1:**
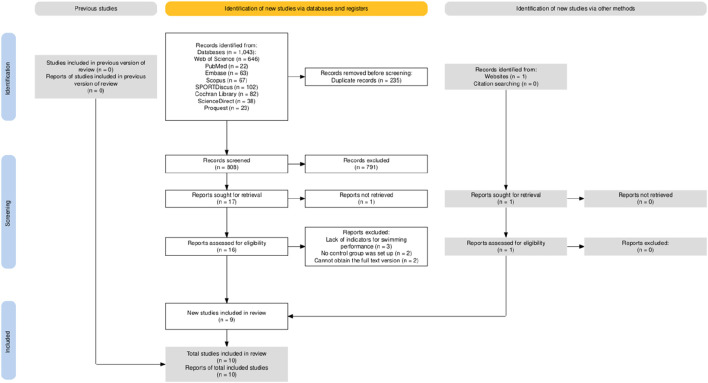
Article retrieval and filtering flow.

The flow diagram illustrates the complete process of the systematic literature search conducted for this study (Figure 1). A list of excluded full-text studies with reasons for exclusion is provided in Supplementary Appendix F. A total of 1,043 records were identified through database searching, and after sequential screening, nine articles (Ohya et al., 2021; Kilding et al., 2010; Wells et al., 2005; Lemaitre et al., 2013; Kapus, 2013; Tan et al., 2023; Lomax et al., 2019; Yañez-Sepulveda et al., 2021; Bernhardt, 2025) met the inclusion criteria. An additional eligible article was identified through other sources (Ghannadi et al., 2024), resulting in 10 studies included in the final analysis (Table 1). The included studies involved 238 participants. After excluding 8 participants from groups with missing follow-up visits, a final total of 230 participants remained (experimental group: n = 117; control group: n = 113). A summary of study characteristics is as follows: six studies included both male and female participants, while four studies included only males. Regarding participant age, four studies included adults, four included minors, and two included both age groups. All 230 participants had prior swimming experience and were not classified as beginners. Regarding swimming performance, seven studies evaluated freestyle events ranging from 50 m to 200 m, while three studies did not specify stroke type. All 10 studies reported intervention duration and training frequency, but only two provided details on the duration of individual training sessions. Nine studies reported training intensity, with most prescribing loads at 50%–80% of MIP; one study did not report intensity. During data extraction, four studies provided image-derived estimates (Ohya et al., 2021; Kilding et al., 2010; Tan et al., 2023; Bernhardt, 2025). Two swimming performance outcomes (Tan et al., 50 m freestyle (Tan et al., 2023); Sepulveda et al., 200 m freestyle (Romer and Polkey, 2008)) were excluded due to significant baseline differences.

**FIGURE 2:**
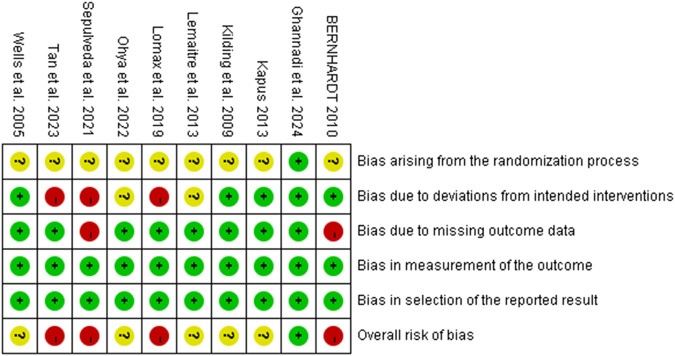
Summary of risk-of-bias judgments across individual studies using the RoB 2 tool. Each domain is evaluated as low risk (green), some concerns (yellow), or high risk (red).

**FIGURE 3 F3:**
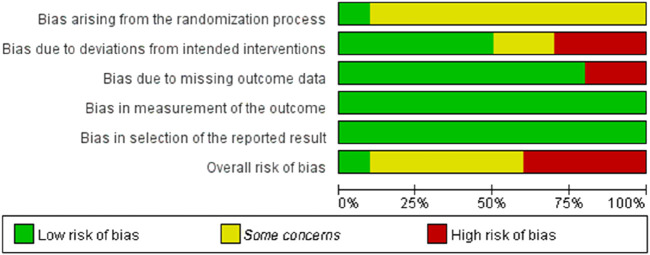
Proportion of included studies rated as low risk, some concerns, or high risk in each RoB 2 domain.

The quality of the research methodology was assessed using the RoB 2 tool. The results are presented in Figures 2,3, [Fig F3], with specific justifications for the judgements provided in Supplementary Appendix C. Of the 10 included studies, overall methodological quality was moderate, with some concerns or high risk of bias noted in several domains. Specifically, most studies were judged as low risk in domains related to outcome measurement and selective reporting. However, concerns were frequently identified in domains such as the randomization process and deviations from intended interventions, and four studies (Tan et al., 2023; Lomax et al., 2019; Yañez-Sepulveda et al., 2021; Bernhardt, 2025) were rated as having a high overall risk of bias due to high risk ratings in one or more domains. In total, five studies (Ohya et al., 2021; Kilding et al., 2010; Wells et al., 2005; Lemaitre et al., 2013; Kapus, 2013) were rated as having “some concerns,” and only one study (Ghannadi et al., 2024) were judged as having an overall low risk of bias. These findings underscore the need for more rigorous study designs in future research, including clearer reporting of randomization procedures and adherence to intervention protocols.

### 3.2 Holistic analysis

**FIGURE 4:**
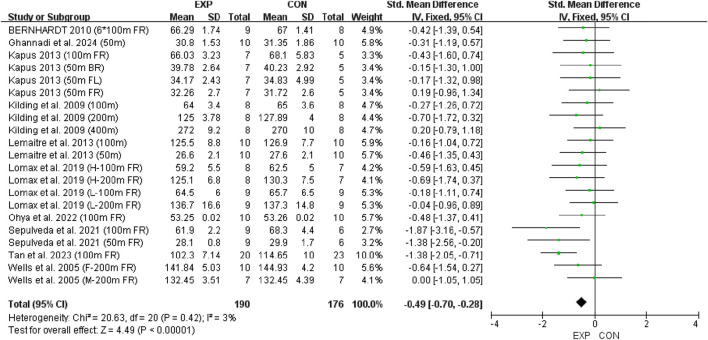
Forest plot showing the overall effect of RMT on swimming performance. Mean = mean, SD = standard deviation, Total = sample size, Std. Mean Difference = standardized mean difference, CI = confidence interval, df = degrees of freedom, IV = inverse variance, Fixed = fixed effects model.

**FIGURE 5:**
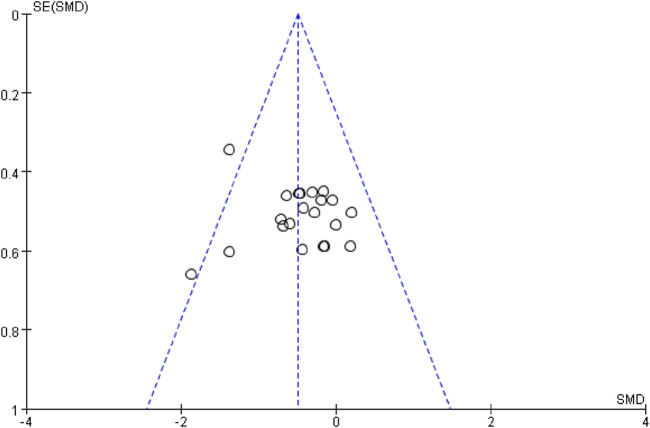
Funnel plot assessing publication bias across included studies. Each dot represents a single outcome; the vertical line indicates the pooled effect, and the dashed lines represent the pseudo 95% confidence limits.

A total of 21 swimming performance outcomes from 10 studies were included in the analysis to assess the pooled effect of RMT on swimming performance. The weighted mean SMD was −0.49 (95% CI [−0.70, −0.28], p < 0.00001; Chi^2^ = 20.63, p = 0.42; I^2^ = 3%), regardless of participants’ gender, age, or intervention type (Figure 4). In this context, shorter completion time indicates better swimming performance. Therefore, the results suggest that RMT has a small but statistically significant effect on swimming performance (0.2 ≤ SMD <0.5), with negligible heterogeneity. Subsequently, a funnel plot was generated (Figure 5) and the Egger’s test was performed, which revealed that the funnel plot was relatively symmetrical and the result of the Egger’s test was p = 0.408 > 0.05, which suggests that there is essentially no publication bias in the included literature.

## 4 Discussion

This meta-analysis is the first to systematically evaluate the effect of RMT on swimming performance. The pooled results revealed a small but significant improvement in performance among swimmers receiving RMT, with low heterogeneity and no publication bias. These findings provide quantitative support for the inclusion of RMT in swim training programs, reinforcing its potential as an effective ergogenic strategy in aquatic sports.

**FIGURE 6:**
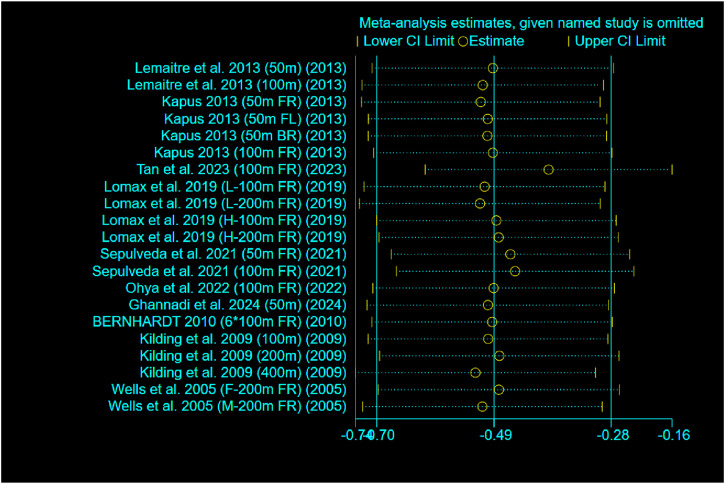
Leave-one-out sensitivity analysis. Each point represents the pooled effect estimate after excluding the named study.

The results of the study showed a small effect size improvement of RMT on swimming performance compared to the control group, with a combined effect size of SMD = −0.49 (95% CI [-0.70, −0.28], p < 0.00001), low heterogeneity (Chi^2^ = 20.63, p = 0.42; I^2^ = 3%) and no significant publication bias (Egger’s test: p = 0.408 > 0.05) further supported this result. In addition, a leave-one-out sensitivity analysis was conducted to evaluate the robustness of the findings (Figure 6). The analysis showed that only the study by Tan et al. exhibited substantial skewness. Excluding this study led to a reduced overall effect size, although it remained within the small effect range (0.2–0.49). This suggests that the exclusion of any single study did not materially alter the overall effect estimate—that is, the beneficial effect of RMT on swimming performance was not driven by any one study. Nevertheless, risk-of-bias considerations temper the confidence in these results. According to the RoB 2 assessment, four included (Tan et al., 2023; Lomax et al., 2019; Yañez-Sepulveda et al., 2021; Bernhardt, 2025) studies were rated as having a high overall risk of bias due to concerns related to deviations from intended interventions and missing outcome data (Figure 2). Although their exclusion did not significantly change the pooled estimate, the potential for bias in these studies cannot be fully dismissed. Therefore, while the meta-analysis supports the positive effects of RMT, the strength of this conclusion should be interpreted with caution given the methodological limitations present across several studies. Future trials should adopt more rigorous designs, including improved randomization, adherence monitoring, and complete outcome reporting, to increase the confidence in effect estimates.

This finding is consistent with the majority of previously published empirical studies. For example, Ohya et al. conducted a 6-week MIP intervention in 30 male swimmers and reported significant improvements in 100 m freestyle performance compared to the control group (HI: p = 0.046; MOD: p = 0.042) (Ohya et al., 2021). However, some studies reported no significant effects of RMT on swimming performance. For instance, Cunha et al. conducted a 12-week intervention involving 32 participants and found no significant changes in swimming performance (p = 0.271), inspiratory muscle strength (p = 0.914), exercise-related lung capacity (p = 0.262), forced expiratory volume in one second (p = 0.265), peak expiratory flow rate (p = 0.270), or perceived dyspnoea (p = 0.568) (Cunha et al., 2019). Despite the overall positive trend, inconsistencies across studies warrant further investigation. Variations in RMT outcomes may be attributed to differences in training load (e.g., 30% vs. 80% of MIP), training frequency (5–14 sessions per week), and intervention duration (4–12 weeks), suggesting the existence of a potential dose–effect relationship. However, only two studies reported detailed training duration (Lemaitre et al., 2013; Tan et al., 2023), limiting our understanding of the optimal training dose. Comparisons were further complicated by the absence of standardised progression protocols (e.g., criteria for increasing training intensity). Future research should clearly define and standardise these parameters. Regarding dose–effect, Mickleborough et al. emphasised that exceeding certain thresholds and maintaining sustained loads are critical for inducing adaptations in pulmonary function, a principle that may also apply to RMT’s performance effects (Mickleborough et al., 2008). Nevertheless, optimal RMT parameters for swimmers remain unclear. With respect to gender, no included study enrolled exclusively female participants, precluding conclusions about potential gender-specific responses to RMT. Variables such as baseline respiratory muscle strength, athletic level (elite vs. sub-elite), age, and intrinsic pulmonary capacity may influence the response to RMT. Yet, the study lacked subgroup stratification or sufficient statistical power to examine these interactions. Lastly, although RMT improves respiratory muscle strength, allocating excessive time to RMT may detract from swimming-specific technical training. Consequently, RMT must be carefully integrated within an athlete’s overall training plan. McConnell emphasized that excessive RMT may compromise sport-specific adaptations if improperly timed or dosed, particularly during periods of high-volume technical training (McConnell, 2013). The concept of a ‘training interference’ underscores the importance of applying the minimum effective dose and aligning RMT with the athlete’s periodized program. This highlights the importance of balancing general physiological adaptation with sport-specific skill acquisition. Given that RMT constitutes an additional training component, its potential to contribute to accumulated fatigue and interfere with sport-specific training adaptations should not be overlooked. Future studies should examine whether integrated periodised programmes combining RMT and swimming training yield superior performance outcomes.

To the authors’ knowledge, this is the first meta-analysis to evaluate the effect of RMT on swimming performance. In a recent meta-analysis focusing solely on lung function in swimmers, Tello et al. reported that RMT significantly improved MIP (MD = 29.35 cmH_2_O; 95% CI [13.04, 45.65] cmH_2_O; p < 0.01), whereas no significant effects were observed for maximal expiratory pressure, forced expiratory volume in one second, or forced vital capacity (Carvajal-Tello et al., 2024). Furthermore, the current findings are broadly consistent with previous meta-analysis investigating the effects of RMT in trained athletes (HajGhanbari et al., 2013; Xavier et al., 2025; León-Morillas et al., 2021; Illi et al., 2012). For example, HajGhanbari et al. reported that RMT had significant positive effects on time-trial performance (MD = 0.40), exercise endurance (MD = 5.17), and Yo-Yo test performance (MD = 4.86) (HajGhanbari et al., 2013). Similar to cycling, swimming imposes high ventilatory demands, and respiratory muscle fatigue may limit blood flow redistribution to working limb muscles, thereby impairing performance (Romer and Polkey, 2008; Harms et al., 2000). RMT may enhance swimming economy by increasing MIP and MEP, delaying respiratory muscle fatigue, and improving oxygen utilization efficiency (Kilding et al., 2010; McConnell, 2009). Notably, the swimming distances included in this study (50–400 m) corresponded to submaximal intensities. The effects of RMT on shorter sprint events (<30 s) may be limited due to their predominant reliance on anaerobic energy pathways (Shei, 2018).

Although the present study observed a consistent trend of improvement across different strokes, the majority of performance outcomes were derived from freestyle events (13 out of 21), which may obscure stroke-specific effects. The lateral breathing pattern in freestyle places high demands on diaphragmatic and intercostal muscle coordination (McLeod, 2009). RMT may improve breathing efficiency by enhancing stroke-breath synchronization, for example, by reducing peak inspiratory resistance during the breathing cycle (Lavin et al., 2015). However, other strokes such as butterfly or breaststroke involve different breathing mechanics, including forward head lifts and varying trunk rotations, which may engage distinct respiratory and postural muscle groups (Barbosa et al., 2006). These biomechanical and muscular differences suggest that the effectiveness of RMT could vary by stroke type. Therefore, future studies should consider evaluating RMT efficacy in stroke-specific contexts and reporting outcomes separately for each stroke. Tailored RMT protocols may be necessary to address the unique ventilatory demands of each swimming style.

## 5 Limitations

Several limitations of this study should be acknowledged. The small number of included studies (n = 10) limited the statistical power of the subgroup analysis. Notably, none of the studies specifically examined female swimmers, precluding any meaningful exploration of gender-based differences. Furthermore, the lack of detailed information on training duration and the absence of standardised progression protocols hindered a robust dose–effect analysis. Although the overall methodological quality was rated as ‘moderate’, four studies were assessed as being at high risk of bias, and most failed to implement adequate blinding and rigorous randomisation procedures. These methodological weaknesses could potentially result in performance bias or an overestimation of the true effects. Additionally, the analysis were unable to adequately account for how RMT was integrated into the swimmers’ overall training programmes, leaving possible confounding effects unresolved. Since all participants had prior swimming experience, the generalisability of the findings to untrained or recreational populations remains limited. Lastly, four studies relied on image-based data (Ohya et al., 2021; Kilding et al., 2010; Tan et al., 2023; Bernhardt, 2025), which may have introduced measurement errors.

## 6 Conclusion

The findings of this meta-analysis suggest that RMT may confer moderate improvements in swimming performance compared to control conditions (SMD = −0.49). RMT serves as a supplementary modality to resistance training in swimmers. Among the included studies, RMT protocols were generally implemented at intensities ranging from 50% to 80% of MIP, over 6–8 weeks, with a training frequency of 3–14 sessions per week. However, due to considerable heterogeneity in programme design and the absence of direct comparisons across RMT modalities, no definitive conclusions can be drawn regarding the optimal training parameters. Moreover, the physiological mechanisms underlying the observed performance benefits remain speculative, as they were not directly assessed within the scope of this review. Future research should aim to clarify the dose-effect relationship, explore long-term physiological adaptations, and evaluate how individual swimmer characteristics—such as sex, stroke style, and competitive level—moderate the effects of RMT.

## Data Availability

The original contributions presented in the study are included in the article/Supplementary Material, further inquiries can be directed to the corresponding author.

## Abbreviations

BR, Breaststroke; FL, Butterfly; FR, Freestyle; CI, Confidence Interval; RMT, respiratory muscle training; IMT, inspiratory muscle training; EMT, expiratory muscle training; MIP, maximal inspiratory pressure; RCT, randomized controlled trial; CCT, controlled clinical trial; ES, effect size; IV, Inverse Variance; SMD, standardized mean difference; PRISMA, Preferred Reporting Items for Systematic Reviews and Meta-analyses; PROSPERO, International Prospective Register of Systematic Reviews.
