# Supplementary material for: The effect of respiratory muscle training on swimming performance: a systematic review and meta-analysis
Source: Front Physiol. 2025 Jul 17;16:1638739. doi: 10.3389/fphys.2025.1638739 (PMC12310600; doi:10.3389/fphys.2025.1638739)
Supplement: Supplementary file 1 [file Supplementaryfile1.docx]

**Appendix A Database Search Terms**

**Web of Science**

(TS=(‘Breathing Exercises’ OR ‘Exercise, Breathing’ OR ‘Respiratory Muscle Training’ OR ‘Muscle Training, Respiratory’ OR ‘Training, Respiratory Muscle’ OR ‘Inspiratory Muscle Training’ OR ‘Expiratory Muscle Training’)) AND TS=(‘Swimming Exercise’ OR ‘Swimming Performance’ OR Swimming)

**ScienceDirect**

(‘Breathing Exercises’ OR ‘Respiratory Muscle Training’ OR ‘Muscle Training, Respiratory’ OR ‘Training, Respiratory Muscle’ OR ‘Inspiratory Muscle Training’ OR ‘Expiratory Muscle Training’) AND (‘Swimming Exercise’ OR ‘Swimming Performance’ OR Swimming)

**Proquest**

subject(‘Breathing Exercises’ OR ‘Exercise, Breathing’ OR ‘Respiratory Muscle Training’ OR ‘Muscle Training, Respiratory’ OR ‘Training, Respiratory Muscle’ OR ‘Inspiratory Muscle Training’ OR ‘Expiratory Muscle Training’) AND subject(‘Swimming Exercise’ OR ‘Swimming Performance’ OR Swimming)

**PubMed**

(((((((‘Breathing Exercises’[MeSH Major Topic]) OR (‘Exercise, Breathing’[MeSH Major Topic])) OR (‘Respiratory Muscle Training’[MeSH Major Topic])) OR (‘Muscle Training, Respiratory’[MeSH Major Topic])) OR (‘Training, Respiratory Muscle’[MeSH Major Topic])) OR (‘Inspiratory Muscle Training’[MeSH Major Topic])) OR (‘Expiratory Muscle Training’[MeSH Major Topic])) AND (((‘Swimming Exercise’[MeSH Major Topic]) OR (‘Swimming Performance’[MeSH Major Topic])) OR (Swimming[MeSH Major Topic]))

**Embase**

('breathing exercises' OR 'exercise, breathing' OR 'respiratory muscle training' OR 'muscle training, respiratory' OR 'training, respiratory muscle' OR 'inspiratory muscle training' OR 'expiratory muscle training') AND ('swimming exercise' OR 'swimming performance' OR swimming)

**Scopus**

TITLE-ABS-KEY ( 'breathing AND exercises' OR 'exercise, AND breathing' OR 'respiratory AND muscle AND training' OR 'muscle AND training, AND respiratory' OR 'training, AND respiratory AND muscle' OR 'inspiratory AND muscle AND training' OR 'expiratory AND muscle AND training' ) AND TITLE-ABS-KEY ( 'swimming AND exercise' OR 'swimming AND performance' OR swimming )

**Cochrane Library**

ID Search Hits

#1 Breathing Exercises 4914

#2 Exercise, Breathing 7113

#3 Respiratory Muscle Training 3962

#4 Muscle Training, Respiratory 3962

#5 Training, Respiratory Muscle 3962

#6 Inspiratory Muscle Training 2255

#7 Expiratory Muscle Training 1427

#8 #1 OR #2 OR #3 OR #4 OR #5 OR #6 OR #7 11825

#9 Swimming Exercise 1081

#10 Swimming Performance 694

#11 Swimming 1714

#12 #9 OR #10 OR #11 1714

#13 #8 AND #12 in Trials 82

**SPORTDiscus**

(‘Breathing Exercises’ OR ‘Exercise, Breathing’ OR ‘Respiratory Muscle Training’ OR ‘Muscle Training, Respiratory’ OR ‘Training, Respiratory Muscle’ OR ‘Inspiratory Muscle Training’ OR ‘Expiratory Muscle Training’) AND (‘Swimming Exercise’ OR ‘Swimming Performance’ OR Swimming)
