# Supplementary figures and images for: The effect of respiratory muscle training on swimming performance: a systematic review and meta-analysis
Source: Front Physiol. 2025 Jul 17;16:1638739. doi: 10.3389/fphys.2025.1638739 (PMC12310600; doi:10.3389/fphys.2025.1638739)

**Appendix C Correlation Chart**

**Egger’s test**


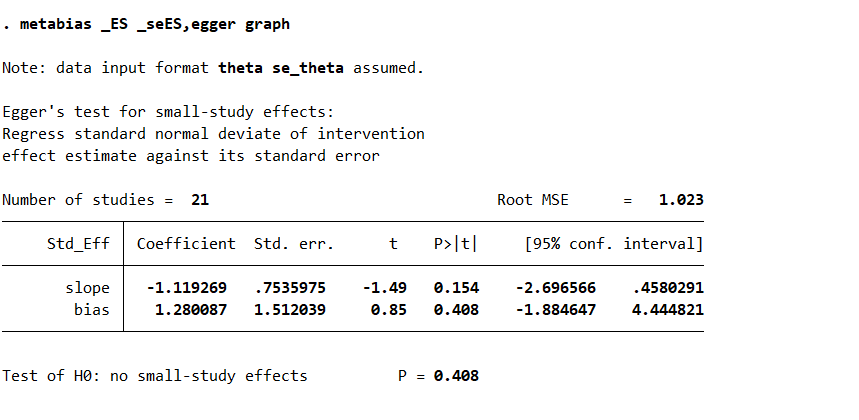

Supplement: Supplementary file 2 [file Supplementaryfile3.docx]
