# Supplementary material for: The effect of respiratory muscle training on swimming performance: a systematic review and meta-analysis
Source: Front Physiol. 2025 Jul 17;16:1638739. doi: 10.3389/fphys.2025.1638739 (PMC12310600; doi:10.3389/fphys.2025.1638739)
Supplement: Supplementary file 3 [file Supplementaryfile2.docx]

**Appendix B Cochrane risk-of-bias tool for randomized trials (RoB 2)**

**Author(s): Shunfang Liu**
**Date:** 2025-07-5

| Author | Year | Bias arising from the randomization process | | Bias due to deviations from intended interventions | | Bias due to missing outcome data | | Bias in measurement of the outcome | | Bias in selection of the reported result | | Overall risk of bias |
| --- | --- | --- | --- | --- | --- | --- | --- | --- | --- | --- | --- | --- |
|  |  | risk-of-bias judgement | Reasons for judgement | risk-of-bias judgement | Reasons for judgement | risk-of-bias judgement | Reasons for judgement | risk-of-bias judgement | Reasons for judgement | risk-of-bias judgement | Reasons for judgement |  |
| Kilding et al. | 2009 | Some concerns | Randomization method was not described, leading to some concerns about allocation concealment. | Low | No significant intervention deviations occurred during the course of the experiment; study participants were blinded and staff were not blinded but followed the designated intervention protocol; and the method of analysis was intentional. | Low | Data for this outcome were available for all, or nearly all, participants randomized or less than 5% of data were missing with balanced dropout. | Low | Measures of outcomes between intervention groups were appropriate, consistent and objective, with little assessor influence. | Low | All outcomes listed in the protocol were reported. | Some concerns |
| Lemaitre et al. | 2013 | Some concerns | Randomization method was not described, leading to some concerns about allocation concealment. | Some concerns | Blind methods are not implemented and intervention deviations may occur. | Low | Data for this outcome were available for all, or nearly all, participants randomized or less than 5% of data were missing with balanced dropout. | Low | Measures of outcomes between intervention groups were appropriate, consistent and objective, with little assessor influence. | Low | All outcomes listed in the protocol were reported. | Some concerns |
| Kapus | 2013 | Some concerns | Randomization method was not described, leading to some concerns about allocation concealment. | Low | No significant intervention deviations occurred during the course of the experiment; study participants were blinded and staff were not blinded but followed the designated intervention protocol; and the method of analysis was intentional. | Low | Data for this outcome were available for all, or nearly all, participants randomized or less than 5% of data were missing with balanced dropout. | Low | Measures of outcomes between intervention groups were appropriate, consistent and objective, with little assessor influence. | Low | All outcomes listed in the protocol were reported. | Some concerns |
| Tan et al. | 2023 | Some concerns | Randomization method was not described, leading to some concerns about allocation concealment. | High | Blinding was not performed and baseline imbalances between different groups are likely to result in intervention bias. | Low | Data for this outcome were available for all, or nearly all, participants randomized or less than 5% of data were missing with balanced dropout. | Low | Measures of outcomes between intervention groups were appropriate, consistent and objective, with little assessor influence. | Low | All outcomes listed in the protocol were reported. | High |
| Lomax et al. | 2019 | Some concerns | Randomization method was not described, leading to some concerns about allocation concealment. | High | Blinding was not performed and baseline imbalances between different groups are likely to result in intervention bias. | Low | Data for this outcome were available for all, or nearly all, participants randomized or less than 5% of data were missing with balanced dropout. | Low | Measures of outcomes between intervention groups were appropriate, consistent and objective, with little assessor influence. | Low | All outcomes listed in the protocol were reported. | High |
| Sepulveda et al. | 2021 | Some concerns | Randomization method was not described, leading to some concerns about allocation concealment. | High | Blinding was not performed and baseline imbalances between different groups are likely to result in intervention bias. | High | Over 15% missing outcome data without sensitivity analysis. | Low | Measures of outcomes between intervention groups were appropriate, consistent and objective, with little assessor influence. | Low | All outcomes listed in the protocol were reported. | High |
| Ohya et al. | 2022 | Some concerns | Randomization method was not described, leading to some concerns about allocation concealment. | Some concerns | Blind methods are not implemented and intervention deviations may occur. | Low | Data for this outcome were available for all, or nearly all, participants randomized or less than 5% of data were missing with balanced dropout. | Low | Measures of outcomes between intervention groups were appropriate, consistent and objective, with little assessor influence. | Low | All outcomes listed in the protocol were reported. | Some concerns |
| BERNHARDT | 2010 | Some concerns | Randomization method was not described, leading to some concerns about allocation concealment. | Low | No significant intervention deviations occurred during the course of the experiment; study participants were blinded and staff were not blinded but followed the designated intervention protocol; and the method of analysis was intentional. | High | Over 15% missing outcome data without sensitivity analysis. | Low | Measures of outcomes between intervention groups were appropriate, consistent and objective, with little assessor influence. | Low | All outcomes listed in the protocol were reported. | High |
| Ghannadi et al. | 2024 | Low | Adequate random sequence generation and concealment described. | Low | No significant intervention deviations occurred during the course of the experiment; study participants were blinded and staff were not blinded but followed the designated intervention protocol; and the method of analysis was intentional. | Low | Data for this outcome were available for all, or nearly all, participants randomized or less than 5% of data were missing with balanced dropout. | Low | Measures of outcomes between intervention groups were appropriate, consistent and objective, with little assessor influence. | Low | All outcomes listed in the protocol were reported. | Low |
| Wells et al. | 2005 | Some concerns | Randomization method was not described, leading to some concerns about allocation concealment. | Low | No significant intervention deviations occurred during the course of the experiment; study participants were blinded and staff were not blinded but followed the designated intervention protocol; and the method of analysis was intentional. | Low | Data for this outcome were available for all, or nearly all, participants randomized or less than 5% of data were missing with balanced dropout. | Low | Measures of outcomes between intervention groups were appropriate, consistent and objective, with little assessor influence. | Low | All outcomes listed in the protocol were reported. | Some concerns |
