# Supplementary material for: The effect of respiratory muscle training on swimming performance: a systematic review and meta-analysis
Source: Front Physiol. 2025 Jul 17;16:1638739. doi: 10.3389/fphys.2025.1638739 (PMC12310600; doi:10.3389/fphys.2025.1638739)
Supplement: Supplementary file 4 [file Supplementaryfile4.docx]

List of excluded studies

| Author | Title | Reasons for exclusion |
| --- | --- | --- |
| Germans Jakubovskis et al. (2024) | Effects of breathing exercises on young swimmers’ respiratory system parameters and performance | Lack of sufficient data for calculating effect sizes |
| Eloi Go´mez-Albareda et al. (2023) | Inspiratory muscle training improves maximal inspiratory pressure without increasing performance in elite swimmers | No control group was set |
| Marisa Cunha et al. (2019) | The effect of inspiratory muscle training on swimming performance, inspiratory muscle strength, lung function, and perceived breathlessness in elite swimmers: a randomized controlled trial | Lack of sufficient data for calculating effect sizes |
| Ray Andrew D. et al. (2008) | Respiratory muscle training against a resistance improves respiratory and underwater swimming performance: 2110: board# 81 May 30 8: 00 am-9: 30 am | The study design was a before and after control |
| Emma E Wilson et al. (2014) | Respiratory muscle specific warm-up and elite swimming performance | Lack of sufficient data for calculating effect sizes |
| Pablo Troncoso Galleguillos et al. (2021) | Efecto del entrenamiento muscular inspiratorio sobre el tiempo de nado y función pulmonar en jóvenes nadadores de alto rendimiento | The study design was a before and after control |
| Sivaguru Muthusamy et al. (2021) | Effects of individualized training and respiratory muscle training in improving swimming performance among collegiate swimmers - an experimental study | Lack of sufficient data for calculating effect sizes |
